# Metformin alleviates inflammatory response and severity rate of COVID-19 infection in elderly individuals

**DOI:** 10.1038/s41598-025-96294-y

**Published:** 2025-04-02

**Authors:** Xuguang Chen, Shengyi Shi, Hanwen Sun, Lei Zhou, Heng Wang, Yan Li, Eric Gilson, Yiming Lu, Lan Hu, Jing Ye

**Affiliations:** 1 Department of Geriatrics, Medical Center On Aging of Shanghai Ruijin Hospital, Shanghai Jiaotong University School of Medicine, Shanghai, China; 2 IRP, Pôle Sino-Français de Recherches en Sciences du Vivant Et Génomique, RuiJin Hospital, Shanghai Jiao Tong University School of Medicine, CNRS, Inserm, Université Côte d’Azur, Shanghai, China; 3 Emergency Department, Shanghai Ruijin Hospital, Shanghai Jiaotong University School of Medicine, Shanghai, China; 4 Inserm, CNRS, IRCAN, Department of Medical Genetics, IHU RespirERA, CHU, Université Côte d’Azur, Nice, France

**Keywords:** Metformin, COVID-19, Elderly, Type 2 diabetes mellitus, Drug development, Viral infection, Respiratory tract diseases, Drug delivery

## Abstract

To evaluate the relationship between metformin and the outcome of coronavirus disease 2019 (COVID-19) infection. The study included 413 patients with type 2 diabetes among the 5217 patients enrolled in a COVID-19 study, and analyzed whether receiving metformin therapy prior to infection was associated with risk of ICU admission, development of pneumonia and length of hospital stay. The study also examined the correlation between metformin treatment and levels of IL-6, CRP, serum ferritin (SF), lymphocyte, CD4 at admission, as well as the increase in open reading frame 1ab gene cycle threshold (ORF1abCT) after one week of hospitalization. There were no differences in age, sex, BMI, comorbidities, number of vaccine doses or eGFR between patients receiving and not receiving metformin therapy. In the ICU group, the proportion of patients not receiving metformin was 92.5%, significantly higher than the 69.2% of patients not admitted to ICU (*p* = 0.010). In the pneumonia group, the proportion of patients not receiving metformin was 78.6%, significantly higher than the 67.2% in the non-pneumonia group (*p* = 0.020). Compared with patients receiving no treatment, those receiving metformin had a shorter hospital stay (12.1 ± 5.9 days vs. 14.5 ± 8.2 days, p = 0.001). In the patients ≥ 60 years old, those receiving treatment had significantly lower levels of IL-6 (median, 12.3 pg/ml vs. 4.0 pg/ml, p = 0.026) and significantly higher levels of Lymphocyte (median, 1.2 × 10^9^/L vs. 1.4 × 10^9^/L, p = 0.015) compared with those not receiving treatment. However, for the patients under 60, there were no significant differences observed in IL-6 and Lymphocyte levels between those receiving treatment and those not. Metformin can reduce the severity of COVID-19 infection and attenuate the inflammatory response associated with COVID-19 infection.

Currently, metformin (MET) is the first-line drug for treating type 2 diabetes mellitus (TDM) in most guidelines used by over 200 million patients every day. It is important to note that MET is a pleiotropic drug whose beneficial effects extend far beyond its impact on glycemic homoeostasis^1^. MET repurposing is being extended to a variety of pathophysiological conditions in a growing body of preclinical and clinical data, including cancer prevention and/or treatment^2^, neurodegenerative diseases^3^, anti-aging^4,5^, cardiovascular disease^6^, chronic obstructive pulmonary disease (COPD)^7^ and polycystic ovary syndrome (PCOS)^8^. Through both AMP activated protein kinase (AMPK)-dependent and AMPK-independent mechanisms, MET also exhibits anti-inflammatory properties and immunomodulatory functions. This pleiotropic profile makes MET a good drug candidate for mitigating the severity of coronavirus disease 2019 (COVID-19)^9^.

Covid-19 has spread throughout the world from year 2020 to 2022 by coronavirus 2 (SARS-CoV-2). During the three years of the pandemic, several retrospective observational studies showed that MET could have both a protective and therapeutic role against COVID-19. For example, MET was found to have the potential to reduce in-hospital mortality and adverse events in patients infected with COVID-19 through mechanisms such as lowering blood glucose, reducing endothelial damage, promoting repair of injured lung tissue, antiviral and immunomodulation^10–13^ However, other studies have shown that MET has no clinical benefit for the early treatment of COVID-19. Therefore, whether MET treatment can ultimately improve the prognosis of COVID-19, remains unresolved.

Between 1 March and 2 July 2022, a total of 649,657 cases of COVID-19 infection were reported in Shanghai, most of which were infected with Omicron BA.2.2 Variants^14,15^. Of these, 413 were diabetic and were used in this study to analyze the putative link between MET treatment and the severity of COVID-19 infection`. We found that MET treatment can reduce inflammation and increase immunity in COVID 19 infection in patients over 60, and has a strong inhibitory effect on IL-6 production. Our results also confirmed that MET has a particular protective function in the elderly and is a potential controller of IL-6 under various conditions, even in viral infection. We discuss these results in the light of MET anti-aging properties.

## Methods

COVID-19 is a respiratory disease caused by SARS-CoV-2, which can be diagnosed by a positive PCR test for SARS-CoV-2 or by clinical and radiographic findings. Mild COVID-19 refers to mild symptoms without evidence of pneumonia on imaging; moderate COVID-19 refers to clinical symptoms with evidence of pneumonia on imaging; severe COVID-19 is defined as a respiratory rate ≥ 30 breaths per minute, oxygen saturation ≤ 93%, oxygenation index ≤ 300 mmHg, and/or lung infiltrates increasing > 50% within 24–48 h; critical COVID-19 is defined as respiratory failure requiring mechanical ventilation, shock, and/or multiorgan dysfunction. Diabetes: the diagnosis of diabetes can be confirmed by medical records documenting a diagnosis of diabetes or reviewed by an endocrinologist. According to the World Health Organization criteria, diabetes can be diagnosed with a fasting plasma glucose ≥ 7.0 mmol/L (≥ 126 mg/dL) or a 2-h plasma glucose ≥ 11.1 mmol/L (≥ 200 mg/dL). Estimated glomerular filtration rate (eGFR): eGFR is calculated using the modified MDRD equation. eGFR is used to assess kidney function, with higher values indicating better kidney function. Medication: Medication use was defined as continuous use of medication in the 3 months prior to COVID-19 infection. This definition was used to avoid confounding effects of medication discontinuation in hospitalized patients. ORF1ab CT: the cycle threshold (Ct) values for amplification of ORF1ab (the open reading frames 1ab, ORF1ab) from individuals infected with Omicron. ΔORF1ab CT: ΔORF1ab CT refers to the difference between the ORF1ab CT at 1 week after admission and the ORF1ab CT at admission. This difference is used to assess the progression or improvement of the disease. Discharge criteria: Discharge criteria include normal body temperature for > 3 days, improvement of respiratory symptoms, significant improvement in chest imaging of acute infiltrative lesions, and two consecutive negative nucleic acid tests on respiratory samples at least 24 h apart.

Primary outcome: The primary outcome is the ICU admission rate, which refers to the proportion of patients requiring transfer to the intensive care unit for treatment.

Secondary outcomes: Secondary outcomes include viral clearance rate, development of pneumonia, and length of hospital stay, which are used to evaluate the treatment response and disease progression in patients.

This retrospective cohort study included 5217 patients aged between 18 and 102 years who were admitted to the North Campus of Ruijin Hospital, affiliated with Shanghai Jiao Tong University School of Medicine, from March 20, 2022, to June 18, 2022. This hospital is one of the designated treatment centers for COVID-19 patients in Shanghai, handling moderate, severe, or critical cases. The patients included in this study were transferred from the community or Fangcang shelter hospitals, which are basic medical facilities converted from public spaces to handle asymptomatic cases or low-risk patients with mild symptoms. All clinical investigations were conducted in accordance with the principles of the Helsinki Declaration. All participants provided written informed consent for the collection and use of test data for research purposes.

Epidemiological, demographic, clinical, laboratory, treatment-related, and outcome-related data were extracted from electronic medical records using standardized data collection forms by experienced clinicians.

In summary, the clinical diagnosis of COVID-19 was made based on the presence of symptoms, exposure, and characteristic lung imaging features consistent with coronavirus pneumonia. As mentioned earlier, SARS-CoV-2 infection was detected in respiratory specimens using second-generation sequencing or real-time fluorescence RT-PCR. Patients underwent routine laboratory tests, such as blood tests, coagulation tests, myocardial enzyme tests, and biochemical tests (including liver and kidney function, blood glucose, and electrolytes). Chest imaging examinations were performed using CT. All patients received standardised antiviral and hormonal therapy in accordance with the Diagnostic and Treatment Protocol for Novel Coronavirus Pneumonia (Trial Ninth Edition) issued by the National Healthcare Commission of China. Clinical outcomes were evaluated by experienced clinicians.

### Statistical analysis

Statistical analysis was performed using different methods. For categorical variables, chi-square test or Fisher’s exact test was used for comparison. For continuous and univariate variables, independent sample t-test or F-test was used for comparisons. For non-univariate variables, non-parametric test (Mann Whitney test) was used for comparison. Logistic regression analysis was used to determine factors associated with clinical outcomes. The correlation of the multiple variables to age was tested using scatter plot analysis and Pearson correlation test. Statistical significance was usually defined as a *P*-value < 0.05. Statistical analysis was conducted using SPSS software.

## Results

### Patient characteristics

**Fig. 1:**
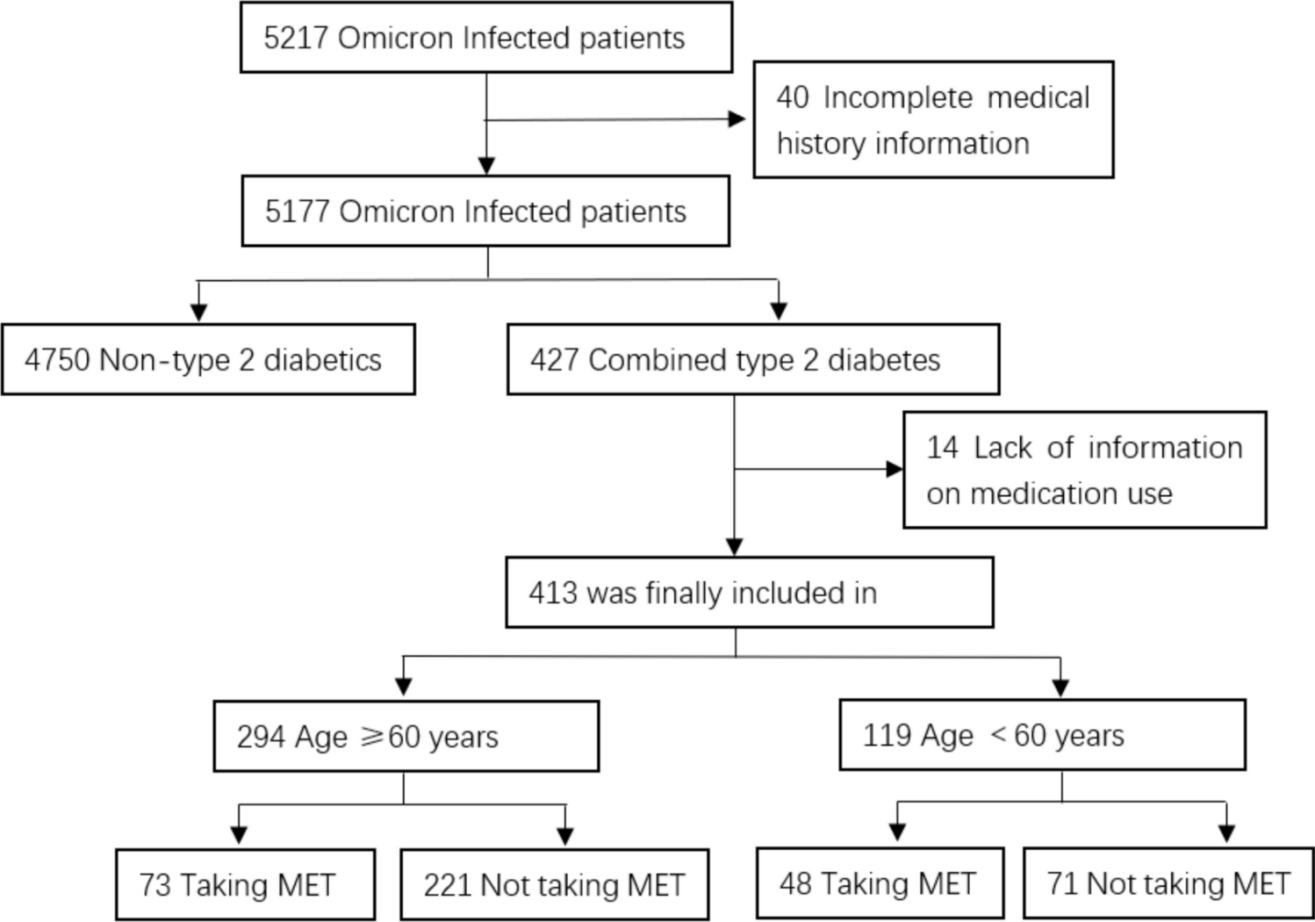
The enrollment strategies of the study. The number of patients were diagnosed and enrolled to the designed groups from March 20, 2022 to June 18, 2022.

A total of 413 patients with type 2 diabetes melitus (TDM) with a mean age of 66.3 ± 13.9 years were included in this study from a cohort of 5217 cases infected with COVID-19 (Fig. 1). Of these, 287 (69.5%) had at least one comorbidity, including coronary heart disease in 79 (19.1%) cases, hypertension in 210 (50.8%) cases, stroke in 65 (15.7%) cases, chronic kidney disease in 41 (9.9%) cases, chronic lung disease in 7 (1.7%) cases, malignancy in 24 (5.8%) cases, and immunodeficiency in 14 (3.4%) cases. 150 (36.3%) cases of patients have received at least one dose of COVID-19 vaccine. Prior to admission, 121 (29.3%) patients were taking MET, 34 were taking sulfonylureas, 13 were taking gliptins, 10 were taking insulin sensitizers, 55 were taking alpha-glucosidase inhibitors, 27 were taking DPP-4 inhibitors, 38 were taking SGLT-2 inhibitors, 101 were using insulin, and 25 were not using any antidiabetic medication or had been using medication for less than 3 months. There were no significant differences in age, sex, BMI, comorbidity rates, or vaccine doses between patients who received MET and those who did not (Table 1).

**Table 1:** Baseline characteristics of all those treated versus not treated with Met.

	Total (N = 413)	MET group (N = 121)	Non-MET group (N = 292)	Z/F	*P*
Age (years)	66.3 ± 13.9	64.4 ± 13.1	67.1 ± 14.2	1.167	0.077
Male	229 (55.4%)	60 (49.6%)	169 (57.9%)	2.380	0.123
BMI (kg/m^2^)	24.7 ± 4.2	16.6 ± 12.2	18.1 ± 11.5	0.458	0.645
Comorbidities	287 (69.5%)	78 (64.5%)	209 (71.6%)	2.041	0.153
Number of vaccinations	0.9 ± 1.2	1.0 ± 1.3	0.9 ± 1.2	3.214	0.320

BMI, body mass index.

### Comparison of laboratory results between diabetic patients receiving and not receiving MET treatment

According to the experimental results in Table 2, there were no statistically significant differences in eGFR, Glucose, ALT, Albumin, WBC, Haemoglobin, PLT, PCT, HbA1c, Fibrinogen, cTnI, and BNP levels between diabetic patients receiving and not receiving MET treatment on admission. This means that there were no significant differences in these laboratory results between diabetic patients receiving and not receiving MET therapies.

**Table 2:** Comparison of laboratory results between diabetic patients receiving and not receiving MET treatment.

	Total (N = 413)	MET group (N = 121)	Non-MET group (N = 292)	Z/F	*P*
eGFR(ml/min/1.73m^2^)	75 ± 35	78 ± 31	74 ± 36	1.223	0.336
Glucose(mmol/L)	8.8 ± 4.5	8.2 ± 3.5	9.0 ± 4.8	8.128	0.084
ALT (U/L)	28 ± 39	26 ± 30	28 ± 41	0.413	0.568
Albumin (g/L)	37 ± 5	38 ± 5	37 ± 5	2.901	0.484
WBC(× 10^9^/L)	6.19 ± 3.42	6.14 ± 3.12	6.21 ± 3.53	1.255	0.848
Haemoglobin (g/L)	129 ± 23	129 ± 23	129 ± 23	0.006	0.909
PLT(× 10^9^/L)	193 ± 82	201 ± 83	190 ± 82	0.046	0.222
PCT (ng/mL)	2.15 ± 9.37	2.85 ± 14.34	1.89 ± 6.79	1.616	0.592
HbA1c (%)	8.4 ± 2.1	7.8 ± 1.5	8.6 ± 2.2	5.557	0.082
Fibrinogen (g/L)	3.95 ± 1.24	3.85 ± 1.15	3.98 ± 1.28	0.061	0.411
cTnI (pg/mL)	127.2 ± 789.9	179.3 ± 992.3	108.1 ± 704.0	1.080	0.558
BNP (pg/mL)	59 (25,186)	48(24,76)	75(28,250)	-0.398	0.691
ORF1abCT	27.1 ± 7.6	28.1 ± 7.7	26.7 ± 7.5	0.714	0.131

eGFR,estimated glomerular filtration rate; ORF1abCT,open reading frame 1ab gene cycle threshold; NCT,nucleocapsid protein gene cycle threshold; ALT, alanine aminotransferase; WBC, white blood cell; PLT, platelet count; protein; PCT, procalcitonin; HbA1C, hemoglobin A1c; cTnI, cardiac troponin I; BNP,brain natriuretic peptide; ORF1abCT,open reading frame 1ab gene cycle threshold.

### Comparison of clinical outcomes between diabetic patients receiving and not receiving MET treatment

Among the 413 patients with type 2 diabetes, 126 (30.5%) had pulmonary infiltrates on imaging. Of these, 27 received MET treatment, 18 (4.4%) were classified as severe or critical on admission and 9 (2.2%) cases progressing to severe or critical during hospitalization. Of all severe or critical cases, 2 received MET treatment and all were admitted to the ICU. The mean length of hospital stay for all COVID-19 infected patients with type 2 diabetes was 13.8 ± 7.7 days.

**Fig. 2:**
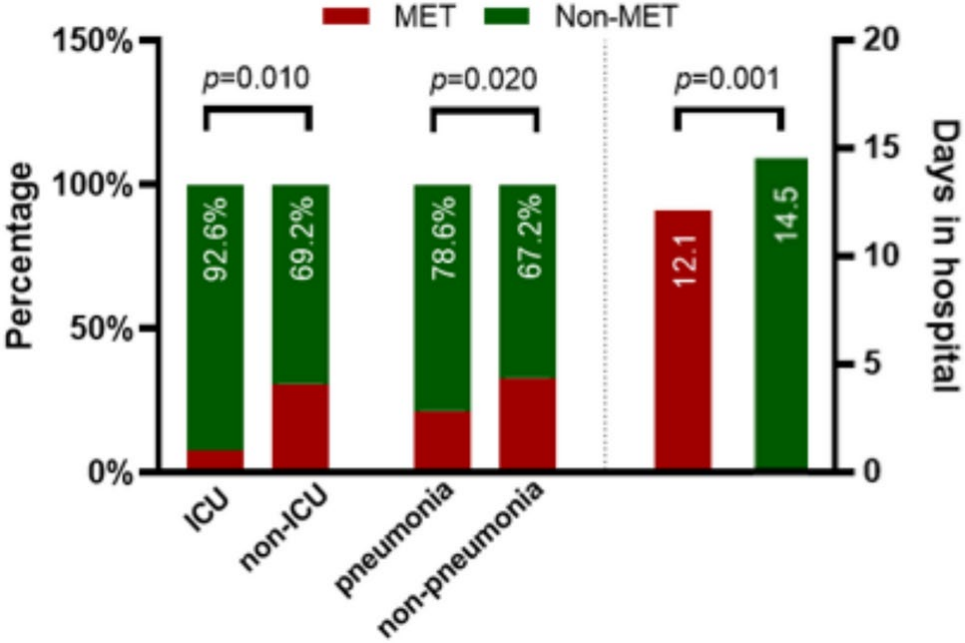
Comparison of clinical outcomes between those treated and not treated with MET among 413 infected patients with type 2 diabetes. Non-DM: Non-type 2 diabetes mellitus group; DM non-MET: Type 2 diabetes mellitus group without MET; DM MET: Type 2 diabetes mellitus group with MET.

Compared with those who received MET treatment, the proportion of patients admitted to the ICU was significantly higher in those who did not receive MET treatment (69.2% vs. 92.6%, *p* = 0.010). Similarly, the proportion of patients with pneumonia was significantly higher in those who did not receive MET treatment than in those who did not have pneumonia (67.2% vs. 78.6%, *p* = 0.020). In addition, patients who received MET treatment had a shorter length of hospital stay than for those who did not receive MET treatment (14.5 ± 8.2 days vs. 12.1 ± 5.9 days, *p* = 0.001) (Fig. 2).

### Comparison between elderly and non-elderly patients receiving and not receiving MET treatment

**Fig. 3:**
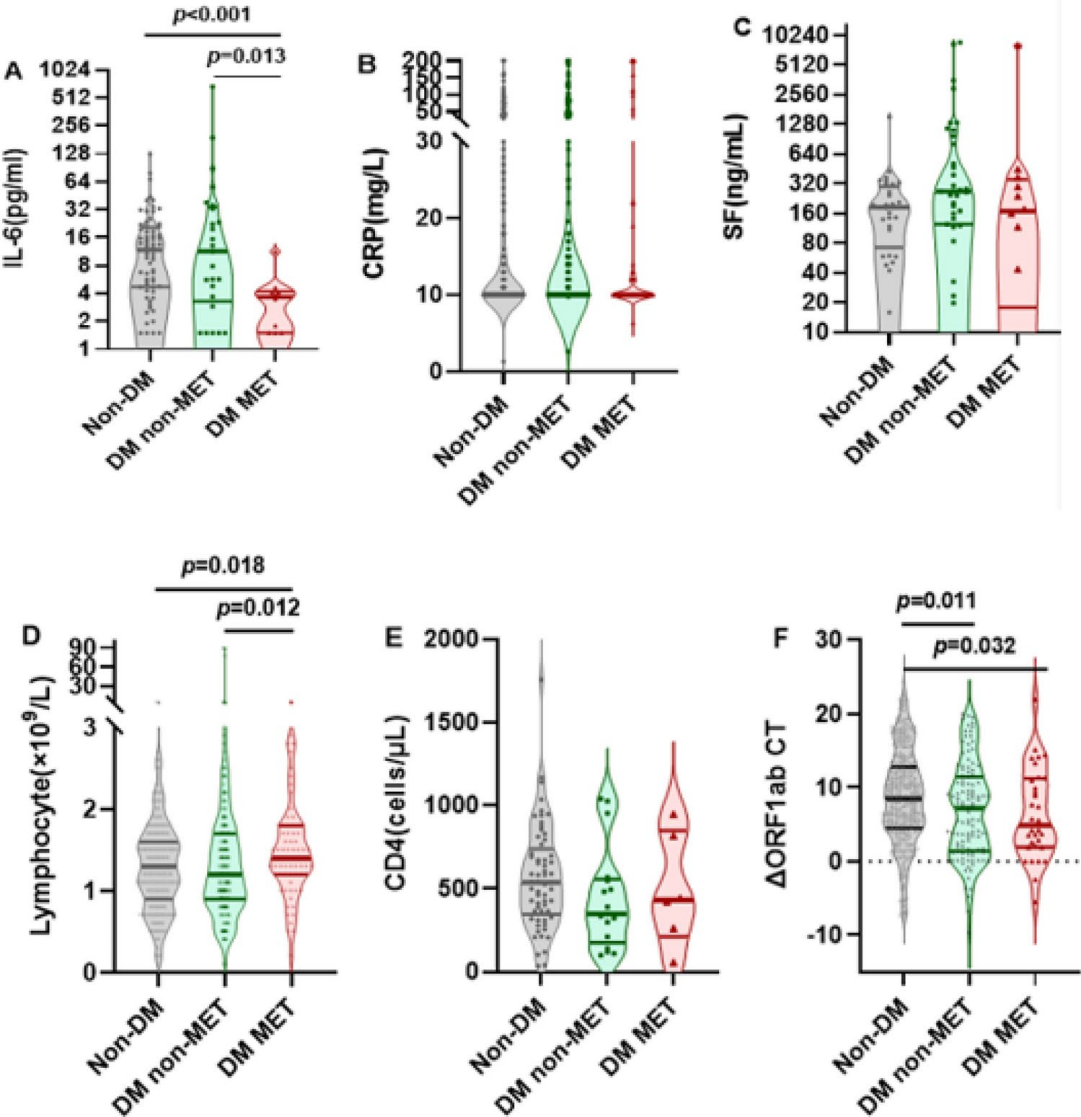
Comparison of levels of inflammatory and immune markers between different groups of infected elderly patients (≥ 60 years). Shown are the level of IL-6 (Panel **A**), CRP (Panel **B**) and SF (Panel **C**), the number of lymphocytes (Panel **D**) and CD4 + cells (Panel E) and the ΔORF1ab CT level (Panel F) in indicated elderly patients (≥ 60 years). Data were analyzed with Mann Whitney test and shown in quartile. Non-DM: Non-type 2 diabetes mellitus group; DM non-MET: Type 2 diabetes mellitus group without MET; DM MET: Type 2 diabetes mellitus group with MET. IL-6,interleukin-6; CRP, c-reactive protein; SF, serum ferritin; ΔORF1ab CT,ORF1ab CT 1 week after admission—ORF1ab CT at admission.

Among patients over 60 years of age (defined as elderly), the proportion of ICU admissions was significantly lower in MET recipients (2.7% vs. 10.4%, p = 0.042), and the length of hospital stay was significantly shorter (12.1 ± 5.5 days vs. 14.9 ± 8.8 days, p = 0.001) (supplementary Table 1). Figure 3 (A-F) demonstrates the differences in levels of inflammation and immune markers between subgroups. The MET-treated patients had significantly lower levels of IL-6 (IQR, 11.4 pg/ml vs. 3.7 pg/ml, p = 0.013; 11.8 pg/ml vs. 3.7 pg/ml, p < 0.001) as compared to the non-diabetic patients of the COVID cohort and those who did not receive MET (Fig. 3A). MET-treated individuals had significantly higher levels of lymphocytes as compared to non-diabetic patients (IQR, 1.3 × 10^9^/L vs. 1.4 × 10^9^/L, p = 0.018) and to those who did not receive MET (IQR, 1.2 × 10^9^/L vs. 1.4 × 10^9^/L, p = 0.012) (Fig. 3D). Compared to non-diabetic patients, both MET-treated individuals and those who did not receive MET had significantly reduced ΔORF1ab CT values (IQR,8.5 vs. 7.2, p = 0.011; 8.5 vs. 4.9, p = 0.032)(Fig. 3F).

**Fig. 4:**
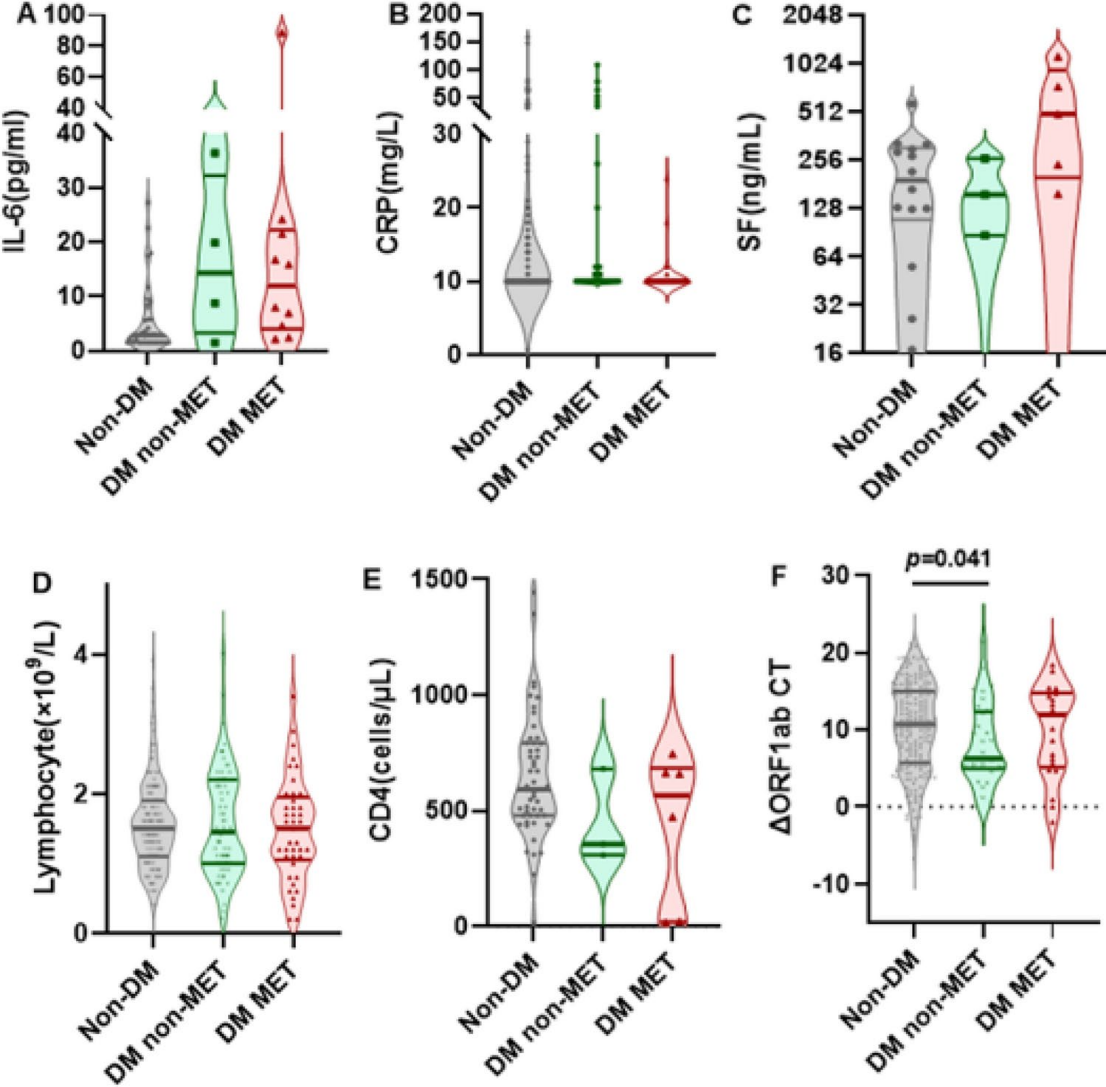
Comparison of levels of inflammatory and immune markers between different groups of infected non-elderly patients (< 60 years). Shown are the level of IL-6 (Panel **A**), CRP (Panel **B**) and SF (Panel **C**), the number of lymphocytes (Panel **D**) and CD4 + cells (Panel **E**) and the ΔORF1ab CT level (Panel **F**) in indicated no-elderly patients (< 60 years). Data were analyzed with Mann Whitney test and shown in quartile. Non-DM: Non-type 2 diabetes mellitus group; DM non-MET: Type 2 diabetes mellitus group without MET; DM MET: Type 2 diabetes mellitus group with MET. IL-6,interleukin-6; CRP, c-reactive protein;SF, serum ferritin; ORF1ab CT: the cycle threshold (Ct) values for amplification of ORF1ab (the expression of the open reading frames 1ab, ORF1ab) from individuals infected with Omicron; ΔORF1ab CT,ORF1ab CT 1 week after admission—ORF1ab CT at admission.

In contrast, for the patients under 60 years of age (defined as non-elderly), there were no significant differences in the risk of ICU admission, pneumonia, and length of hospital stay between MET-treated and non-treated patients (supplementary Table 2). In agreement with this result, none of the inflammation and immunological markers showed significant differences between the MET-treated and non-treated diabetic patients (Fig. 4A–F). Only as compared to non-diabetic individuals, the patients who did not receive MET had significantly increased viral clearance (ΔORF1ab CT, IQR,10.6 vs. 6.3, p = 0.041) (Fig. 4F).

To investigate the possibility that the decrease of inflammatory markers in the elderly patients treated with MET might be due to a slight age and glucose effect, as age and glucose were mildly decreased in the group of receiving MET treatment although not statistically significant (Supplementary Tab1), we analyzed the correlation between IL-6, CRP, SF, lymphocytes, CD4, ΔORF1ab CT and age or glucose. Only ΔORF1ab CT was correlated to age and glucose (Supplementary Table 3), which is consistent with the results that MET treatment did not significantly ameliorate the level of ΔORF1ab CT, and the diabetes patients have a lower ΔORF1ab CT than non-diabetes COVID-19 patients. Moreover, a scatter plot analysis did not reveal a correlation between age, glucose and inflammatory markers in elderly patients (Supplementary Fig. 1). Therefore, we conclude that the beneficial effect of MET on elderly patients is dependent on inflammatory inhibition but independent on age and glucose.

## Discussion

The main result of this study is to show, for a series of diabetic patients aged over 60 and hospitalized for COVID-19 infection, the benefit of MET treatment in terms of ICU admission, pneumonia and number of hospitalization days compared with other anti-diabetic treatments. These findings concur with previous research that MET therapy can increase patients’ chances of discharge within 28 days^16^, reduce ICU admission rate^17^, admission rates^22^, and decrease in-hospital mortality rates^18,19^. A recent randomized trial did not observe a preventive effect of MET on the occurrence of COVID-19-related hypoxemia, emergency room visits, hospitalizations or death^25^. The seemingly discrepancy with our results might be explained by the fact that in our case (diabetic) MET is administered long-term before COVID-19 infection.

As glucose control and BMI were similar in both types of diabetic patients, our results suggest an effect of MET independent of its hypoglycemic effects. On the one hand, many recent studies highlight the anti-aging effects of MET, which is being considered as a preventive treatment for age-related diseases in the general population^20^. On the other hand, the severity of COVID-19 infection is not only linked to age but a consequence of the biological hallmarks of aging, including cellular senescence^21,22^. It is therefore quite possible that the benefits of MET for COVID-19 patients rely on its anti-aging properties. Indeed, we found in MET-treated patients a decrease in inflammatory markers. An anti-inflammatory effect is also found in the study conducted by Yuchen Chen et al^23^ but not observed in another study^19^. We also found an increase in circulating lymphocytes, in concordance with^24^. In line with this interpretation, there is no effect of MET on viral clearance among diabetes patients, suggesting that MET does not act on viral replication.

Another strong argument in favor of an effect of age on the beneficial effect of MET is that patients under 60 years of age do not show a significant benefit from MET, although a slight trend can be observed. Many possible reasons underlie these age‐associated differences, including different cell susceptibility to viruses, existence of underlying chronic diseases, and different immune response and capacity against viral infection. In any case, this difference raises the question of the use of MET in the general non-diabetic population, whether to prevent complications of COVID-19 infection or for an overall anti-aging effect.

COVID-19 infection is associated with a weakened immune response, chronic inflammation and potential direct pancreatic damage^17^. Excessive inflammatory response and immune suppression are important mechanisms by which COVID-19 progresses to severe lung damage. It is therefore possible that, among the various pathways of aging counteracted by MET, it is the anti-inflammatory and immunomodulatory effects that are decisive for its benefit in COVID-19 patients^18–20^. Noteworthy, animal experiments have shown that MET can inhibit NLRP3 inflammasome activation by suppressing mitochondrial ATP and DNA synthesis, protecting mice against LPS-induced acute respiratory distress syndrome (ARDS) and attenuating pulmonary inflammation in COVID-19-infected mice^25^. This may explain the observed reduction in the risk of pneumonia with MET treatment, and suggests that MET may contribute to attenuating the severity of COVID-19 infection.

The results of this study reinforce the value of senotherapies, not only for diabetics but also for elderly subjects in the general population.

## Limitations

Due to the lack of information on the dosage of MET taken, we cannot determine the impact of dosage on outcomes. Not all patients received CT scans, and there were 12 cases of missing chest CT scans in patients transferred from external hospitals with pneumonia. Due to the exploratory nature of the results, our work must be interpreted as post-hoc analysis, and the chosen multivariate methods inferred through design lack control over Type I errors. Specific treatments for COVID-19, such as corticosteroids or antiviral drugs, may have an impact on prognosis, but our study lacks complete data on such treatments administered during hospitalization.

## Conclusion

This study confirms that pre-infection treatment with MET can have a beneficial effect on COVID-19 prognosis. Our result suggest that MET treatment reduces the severity rate of Covid-19 infection by alleviating inflammatory response through decreasing IL-6 level and enhance immunity through increasing lymphocytes. MET has a specific protective function for the elderly.

## Supplementary Information

Supplementary Information.

## Data Availability

The datasets used and/or analysed during the current study available from the corresponding author on reasonable request.
