# Supplementary material for: Metformin alleviates inflammatory response and severity rate of COVID-19 infection in elderly individuals
Source: Sci Rep. 2025 Apr 2;15:11340. doi: 10.1038/s41598-025-96294-y (PMC11965306; doi:10.1038/s41598-025-96294-y)
Supplement: Supplementary file 1 — Supplementary Information. [file 41598_2025_96294_MOESM1_ESM.docx]

**Supplementary material**

**Supplementary Tab 1**

|  | Age ≥ 60（N=294） | MET group（N=73） | Non-MET group（N=221） | Z/F | *P* |
| --- | --- | --- | --- | --- | --- |
| Age (years) | 72.9±9.5 | 71.4±8.3 | 73.5±9.9 | 10.664 | 0.067 |
| BMI（kg/m^2^） | 24.5±4.1 | 24.6±4.1 | 24.5±4.1 | 0.004 | 0.787 |
| Comorbidities | 219(74.5%) | 54(74.0%) | 165(74.7%) | 0.014 | 0.907 |
| Number of vaccinations | 0.7±1.1 | 0.8±1.2 | 0.7±1.1 | 1.388 | 0.552 |
| ICU admission | 25(8.5%) | 2(2.7%) | 23(10.4%) | 4.146 | 0.042 |
| Pneumonia | 108（30.8%） | 21（28.8%） | 87（39.4%） | 2.653 | 0.103 |
| Length of hospitalisation (days) | 13.8±7.7 | 12.1±5.5 | 14.9±8.8 | 11.694 | 0.001 |
| eGFR（ml/min/1.73m^2^） | 71±35 | 73±30 | 71±37 | 1.710 | 0.557 |
| Glucose（mmol/L） | 8.6±4.3 | 8.3±3.6 | 8.7±4.5 | 3.196 | 0.497 |
| ALT（U/L） | 27±42 | 26±34 | 27±45 | 0.056 | 0.818 |
| Albumin（g/L） | 37±5 | 37±5 | 36±5 | 0.438 | 0.303 |
| WBC（×10^9^/L） | 6.29±3.56 | 6.20±3.45 | 6.33±3.61 | 0.633 | 0.794 |
| Haemoglobin（g/L） | 126±22 | 126±24 | 127±21 | 0.631 | 0.718 |
| PLT（×10^9^/L） | 186±84 | 192±83 | 184±85 | 0.016 | 0.498 |
| PCT（ng/mL） | 2.32±10.00 | 3.37±15.61 | 1.95±7.14 | 2.318 | 0.490 |
| HbA1c（%） | 8.3±2.1 | 7.7±1.4 | 8.4±2.3 | 6.449 | 0.103 |
| Fibrinogen（g/L） | 4.01±1.32 | 3.89±1.18 | 4.05±1.37 | 0.328 | 0.376 |
| cTnI（pg/mL） | 150.5±873.3 | 205.7±1078.4 | 129.2±784.0 | 0.820 | 0.604 |
| BNP（pg/mL） | 268±608 | 344±864 | 237±467 | 5.783 | 0.399 |
| ΔORF1ab CT | 3.3±5.3 | 6.4±6.1 | 6.9±6.5 | 2.473 | 0.477 |
| L | 1.3±0.6 | 1.4±0.6 | 1.3±0.6 | 0.625 | 0.171 |
| CRP | 36±49 | 35±43 | 37±51 | 0.413 | 0.776 |
| SF | 265(150,888) | 168(17,353) | 265(124,971) | 5.419 | 0.385 |
| IL-6 | 5.65(1.5,32.4) | 4.0(1.6,9.6） | 12.3(3.5,36.4) | 6.241 | 0.026 |
| CD4 | 692(484,1007) | 882(817,947) | 558(459,1030) | -0.568 | 0.570 |

BMI, body mass index; eGFR,estimated glomerular filtration rate; ORF1abCT,open reading frame 1ab gene cycle threshold; NCT,nucleocapsid protein gene cycle threshold; ALT, alanine aminotransferase; WBC, white blood cell; PLT, platelet count; CRP, c-reactive protein; PCT, procalcitonin; HbA1C, hemoglobin A1c; cTnI, cardiac troponin I; BNP,brain natriuretic peptide; ΔORF1ab CT,ORF1ab CT 1 week after admission - ORF1ab CT at admission; IL-6,[interleukin-6](https://www.bing.com/ck/a?!&&p=8425c1d987c28af5JmltdHM9MTY4MDgyNTYwMCZpZ3VpZD0wNjFlMDBiNi0wMmQ4LTY1OGItMTA5Zi0xMWJmMDM5MjY0MmEmaW5zaWQ9NTE4Mg&ptn=3&hsh=3&fclid=061e00b6-02d8-658b-109f-11bf0392642a&psq=IL-6&u=a1aHR0cHM6Ly9wdWJtZWQubmNiaS5ubG0ubmloLmdvdi8zMTk1ODc5Mi8&ntb=1); CRP, c-reactive protein;SF, serum ferritin; L,Lymphocyte.

**Supplementary Tab 2**

|  | Age ＜ 60（N=119） | MET group（N=48） | Non-MET group（N=71） | Z/F | *P* |
| --- | --- | --- | --- | --- | --- |
| Age (years) | 50.0±8.6 | 49.8±8.7 | 50.1±8.7 | 1.395 | 0.840 |
| BMI（kg/m^2^） | 25.4±4.4 | 26.0±3.1 | 25.3±4.8 | 2.340 | 0.574 |
| Comorbidities | 61（51.3%） | 18（37.5%） | 43（60.1%） | 6.097 | 0.014 |
| Number of vaccinations | 1.4±1.3 | 1.6±1.4 | 1.3±1.3 | 0.033 | 0.336 |
| ICU admission | 2(1.7%) | 0(0%) | 2(2.8%) | 1.375 | 0.241 |
| Pneumonia | 108（30.8%） | 4（10.5%） | 12（17.1%） | 0.854 | 0.355 |
| Length of hospitalisation (days) | 13.0±6.3 | 12.0±7.1 | 13.4±6.0 | 1.874 | 0.319 |
| eGFR（ml/min/1.73m^2^） | 86±33 | 93±30 | 84±33 | 0.303 | 0.246 |
| Glucose（mmol/L） | 9.2±4.9 | 7.7±3.0 | 10±5.5 | 10.343 | 0.043 |
| ALT（U/L） | 30±25 | 25±14 | 31±28 | 1.952 | 0.296 |
| Albumin（g/L） | 40±5 | 40±3 | 40±5 | 3.731 | 0.930 |
| WBC（×10^9^/L） | 5.91±3.00 | 5.94±1.85 | 5.90±3.30 | 0.787 | 0.958 |
| Haemoglobin（g/L） | 137±25 | 139±20 | 136±26 | 2.049 | 0.608 |
| PLT（×10^9^/L） | 211±73 | 230±81 | 205±70 | 0.085 | 0.127 |
| PCT（ng/mL） | 1.03±3.02 | 0.05±0.06 | 1.49±3.61 | 4.043 | 0.350 |
| HbA1c（%） | 9.1±1.7 | 8.6±1.9 | 9.3±1.9 | 0.091 | 0.563 |
| Fibrinogen（g/L） | 3.72±0.88 | 3.73±1.06 | 3.72±0.82 | 0.721 | 0.998 |
| cTnI（pg/mL） | 24.8±62.1 | 35.3±102.5 | 21.7±46.6 | 3.288 | 0.571 |
| BNP（pg/mL） | 206±418 | 276±522 | 182±383 | 1.053 | 0.547 |
| ΔORF1ab CT | 8.9±5.6 | 9.9±6.0 | 8.2±5.3 | 0.002 | 0.060 |
| L | 1.6±0.7 | 1.8±0.7 | 1.5±0.7 | 0.137 | 0.142 |
| CRP | 15±17 | 11±3 | 17±19 | 11.392 | 0.094 |
| SF | 252(157,675) | 496(199,934) | 156(87,263) | -1.640 | 0.101 |
| IL-6 | 12.4(4.1,22.3) | 12.0(4.1,22.3) | 14.4(3.3,32.3) | -0.141 | 0.888 |
| CD4 | 474(164,672) | 566(19,684) | 355(308,681) | 0.000 | 1.000 |

BMI, body mass index; eGFR,estimated glomerular filtration rate; ORF1abCT,open reading frame 1ab gene cycle threshold; NCT,nucleocapsid protein gene cycle threshold; ALT, alanine aminotransferase; WBC, white blood cell; PLT, platelet count; CRP, c-reactive protein; PCT, procalcitonin; HbA1C, hemoglobin A1c; cTnI, cardiac troponin I; BNP,brain natriuretic peptide; ΔORF1ab CT,ORF1ab CT 1 week after admission - ORF1ab CT at admission; IL-6,[interleukin-6](https://www.bing.com/ck/a?!&&p=8425c1d987c28af5JmltdHM9MTY4MDgyNTYwMCZpZ3VpZD0wNjFlMDBiNi0wMmQ4LTY1OGItMTA5Zi0xMWJmMDM5MjY0MmEmaW5zaWQ9NTE4Mg&ptn=3&hsh=3&fclid=061e00b6-02d8-658b-109f-11bf0392642a&psq=IL-6&u=a1aHR0cHM6Ly9wdWJtZWQubmNiaS5ubG0ubmloLmdvdi8zMTk1ODc5Mi8&ntb=1); CRP, c-reactive protein;SF, serum ferritin; L,Lymphocyte.

**Supplementary Tab 3**

|  | Age | | Glu | |
| --- | --- | --- | --- | --- |
|  | r | p | r | p |
| IL-6 | 0.0990 | 0.4987 | 0.1964 | 0.2185 |
| CRP | 0.0818 | 0.1283 | 0.0713 | 0.2203 |
| SF | 0.1275 | 0.3489 | 0.1157 | 0.4236 |
| Lymphocyte | 0.1107 | 0.0307 | 0.0101 | 0.8561 |
| CD4 | 0.3292 | 0.1565 | 0.3301 | 0.1957 |
| ΔORF1ab CT | -0.2128 | 0.0029 | 0.1885 | 0.0170 |

ΔORF1ab CT,ORF1ab CT 1 week after admission - ORF1ab CT at admission; IL-6,[interleukin-6](https://www.bing.com/ck/a?!&&p=8425c1d987c28af5JmltdHM9MTY4MDgyNTYwMCZpZ3VpZD0wNjFlMDBiNi0wMmQ4LTY1OGItMTA5Zi0xMWJmMDM5MjY0MmEmaW5zaWQ9NTE4Mg&ptn=3&hsh=3&fclid=061e00b6-02d8-658b-109f-11bf0392642a&psq=IL-6&u=a1aHR0cHM6Ly9wdWJtZWQubmNiaS5ubG0ubmloLmdvdi8zMTk1ODc5Mi8&ntb=1); CRP, c-reactive protein; SF, serum ferritin


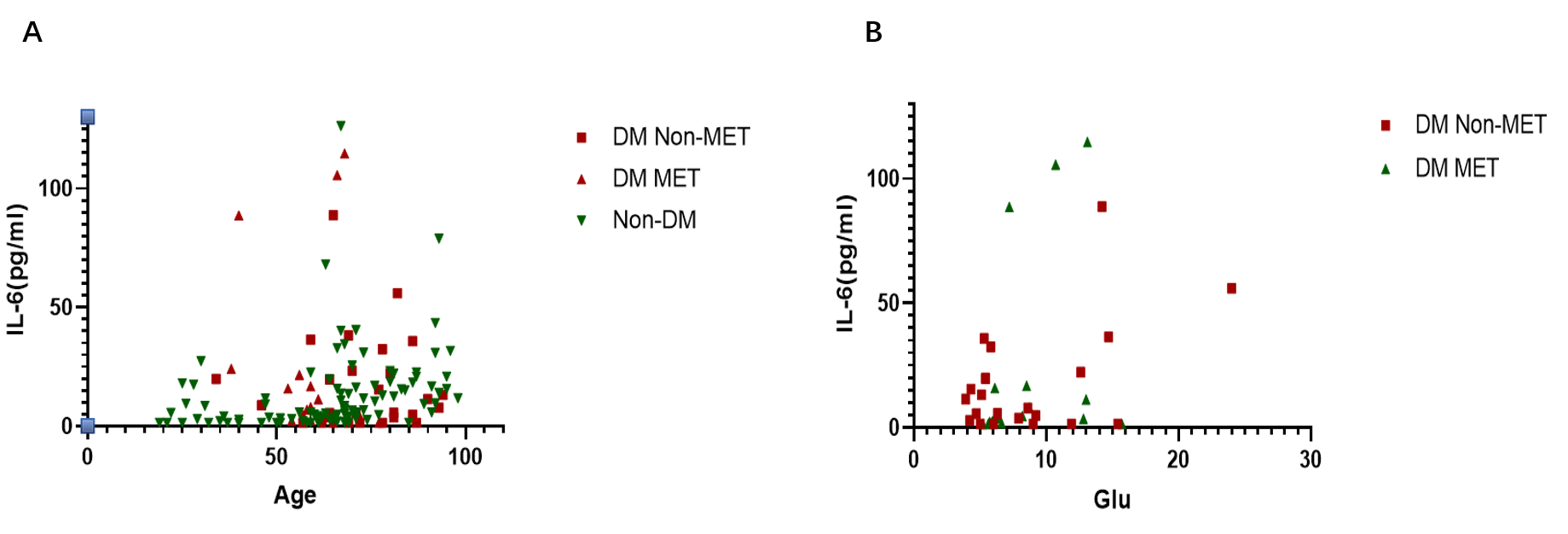


Supplementary Fig 1 The correlation of IL-6 with Age (Panel A) and IL-6 with Glu (Panel B). The analysis was done with scatter plot analysis.

Non-DM: Non-type 2 diabetes mellitus group; DM non-MET: Type 2 diabetes mellitus group without MET; DM MET: Type 2 diabetes mellitus group with MET. The r and P value was shown in Supplementary Tab3.
